# Protective Effects of Thymoquinone, an Active Compound of *Nigella sativa*, on Rats with *Benzo(a)pyrene*-Induced Lung Injury through Regulation of Oxidative Stress and Inflammation

**DOI:** 10.3390/molecules26113218

**Published:** 2021-05-27

**Authors:** Mohammad A. Alzohairy, Amjad Ali Khan, Mohammed A. Alsahli, Saleh A. Almatroodi, Arshad Husain Rahmani

**Affiliations:** 1 Department of Medical Laboratories, College of Applied Medical Sciences, Qassim University, Buraydah 51452, Saudi Arabia; Zhiery@qu.edu.sa (M.A.A.); shly@qu.edu.sa (M.A.A.); smtrody@qu.edu.sa (S.A.A.); 2 Department of Basic Health Sciences, College of Applied Medical Sciences, Qassim University, Buraydah 51452, Saudi Arabia; akhan@qu.edu.sa

**Keywords:** benzopyrene, thymoquinone, antioxidant status, inflammatory markers, immunohistochemistry, TUNEL assay

## Abstract

Benzopyrene [B(a)P] is a well-recognized environmental carcinogen, which promotes oxidative stress, inflammation, and other metabolic complications. In the current study, the therapeutic effects of thymoquinone (TQ) against B(a)P-induced lung injury in experimental rats were examined. B(a)P used at 50 mg/kg b.w. induced lung injury that was investigated via the evaluation of lipid profile, inflammatory markers, nitric oxide (NO), and malondialdehyde (MDA) levels. B(a)P also led to a decrease in superoxide dismutase (SOD) (34.3 vs. 58.5 U/mg protein), glutathione peroxidase (GPx) (42.4 vs. 72.8 U/mg protein), catalase (CAT) (21.2 vs. 30.5 U/mg protein), and total antioxidant capacity compared to normal animals. Treatment with TQ, used at 50 mg/kg b.w., led to a significant reduction in triglycerides (TG) (196.2 vs. 233.7 mg/dL), total cholesterol (TC) (107.2 vs. 129.3 mg/dL), and inflammatory markers and increased the antioxidant enzyme level in comparison with the group that was administered B(a)P only (*p* < 0.05). B(a)P administration led to the thickening of lung epithelium, increased inflammatory cell infiltration, damaged lung tissue architecture, and led to accumulation of collagen fibres as studied through haematoxylin and eosin (H&E), Sirius red, and Masson’s trichrome staining. Moreover, the recognition of apoptotic nuclei and expression pattern of NF-κB were evaluated through the TUNEL assay and immunohistochemistry, respectively. The histopathological changes were found to be considerably low in the TQ-treated animal group. The TUNEL-positive cells increased significantly in the B(a)P-induced group, whereas the TQ-treated group showed a decreased apoptosis rate. Significantly high cytoplasmic expression of NF-κB in the B(a)P-induced group was seen, and this expression was prominently reduced in the TQ-treated group. Our results suggest that TQ can be used in the protection against benzopyrene-caused lung injury.

## 1. Introduction

Lung-associated diseases, including cancer, are one of the major causes of death worldwide. Some common lung diseases are also closely associated with cigarette smoking, which is accountable for the high incidence of lung cancer globally. It has been reported that long-term cigarette smoking can accelerate the lung cancer progression by 10-fold [1]. Benzo(a)pyrene (B(a)P) is one of the principal constituents of tobacco smoke belonging to the polycyclic aromatic hydrocarbon family and performs a key role in the initiation of lung carcinogenesis [2,3,4]. Moreover, the metabolites of B(a)P metabolism produce reactive oxygen species (ROS) and form DNA adducts in cells [5,6]. Furthermore, it has been observed that B(a)P affects the cellular lipid profile in different tissues. In addition, abnormal lipid synthesis or its defective metabolism is implicated in different pathological conditions [7,8].

Various other factors involved in lung pathogenesis include severe air pollution and occupational exposure to dust, fumes, and smoke. The common mediators associated with lung inflammation include interleukin-6 (IL-6), interleukin-1β (IL-1β), and tumour necrosis factor-α (TNF-α). These markers regulate the maturation of dendritic cells as well as promote neutrophil recruitment at inflammatory sites [9,10]. ROS initiates the transcription factors such as nuclear factor-kappa B (NF-κB), which regulates the discharge of pro-inflammatory cytokines such as IL-6 and TNF-α and which promotes the activation of cyclooxygenase-2 [11,12]. 

In spite of great efforts to discover some active pharmacological approaches to the treatment of lung pathogenesis, the mortality rate of this disease is still high. The lung pathogenesis prevention and reduction of the mortality rate due to such diseases seem to be promising through the use of natural compounds which have the least undesirable side effects. In the search of alternative drugs against lung pathogenesis, thymoquinone (TQ), an active compound of black seed (*Nigella sativa*), belonging to the family Ranunculaceae, is a commonly used medicinal plant worldwide. 

The therapeutic role of thymoquinone in different diseases has been reported widely. Its role has been observed in cancer through the suppression of cancer cell stages as well as the activation of some signalling pathways like PI3K/Akt [13]. TQ has also been used as a potent antidiabetic agent by lowering the concentrations of cholesterol and triglycerides, and enhancing the high-density lipoprotein, and insulin sensitivity [14]. In addition, the intraperitoneal administration of thymoquinone in animal models significantly prevents the oxidative damage by enhancing the levels of antioxidant enzymes such as superoxide dismutase and catalase activity [15]. TQ also acts as an anti-inflammatory agent and prevents the expression of IL-6, IL-1β, and cyclooxygenase-2 in experimental rats. Moreover, thymoquinone significantly enhances the concentrations of antioxidant enzymes and lowers the MDA level [16]. 

Thymoquinone has been selected for this study because of its proper scientific evidence against lung pathogenesis treatment. Different doses of TQ have been used in animal models through different routes to evaluate its therapeutic potential against several types of pathogenesis. In a previous study, TQ (50 mg/kg b.w.) was used and it was found to play a significant role in the reduction of pathogenesis [17]. TQ possesses substantial protective effects against oxidative stress mediators such as carbon tetrachloride (CCl_4_)-caused hepatotoxicity [18]. The administration of TQ through oral gavage led to a significant reduction in pro-inflammatory mediators [19], and this compound decreased lipid peroxidation changes [20]. Based on the literature review, the current study was aimed to examine the defensive role of TQ against B(a)P-induced lung injury through the modulation of antioxidant and inflammatory parameters.

## 2. Results 

### 2.1. Effect of Thymoquinone on Lipid Profile Parameters

**Figure 1:**
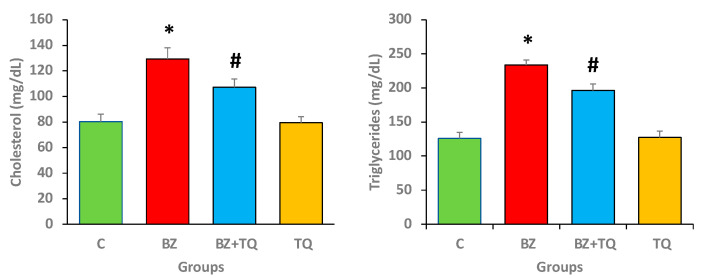
The lipid profile as cholesterol and triglyceride levels in different animal groups. The values indicate the mean ± SEM, with 8 animals/group. The animals administered with B(a)P showed these parameters at significantly higher levels than the control group. The TQ (50 mg/kg b.w.) promoted a significant reduction in lipid profile. The statistical differences are denoted with an asterisk (*), indicating significance at *p* < 0.05 in comparison with the control group, and a hashtag (#), signifying *p* < 0.05 in comparison with the disease control.

The lipid profile was estimated in all experimental animal groups to compare the protective effects of TQ. The disease control animals treated with B(a)P only showed considerably higher triglycerides (233.7 mg/dL) and total cholesterol level (129.3 mg/dL) (*p* < 0.05) than the control group. The TQ at 50 mg/kg b.w. promoted a noteworthy reduction (*p* < 0.05) in these parameters (Figure 1) in comparison with the group administered B(a)P only (*p* < 0.05). This finding clearly evidences that TQ has a lipid-lowering effect.

### 2.2. Effect of TQ on Total Protein and Albumin Levels

**Figure 2:**
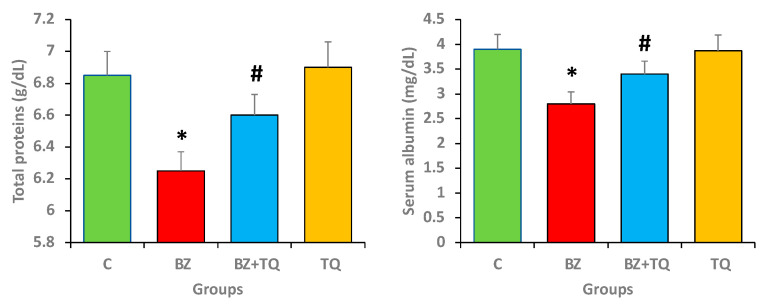
The protein profile (total protein and serum albumin) in different animal groups. The values indicate the mean ± SEM, with 8 animals/group. The statistical differences are denoted with an asterisk (*), indicating significance at *p* < 0.05 in comparison with the control group, and a hashtag (#), signifying *p* < 0.05 in comparison with the disease control group.

The total serum protein as well as albumin levels were estimated in all experimental animal groups to compare the protective effects of TQ and exposure to B(a)P. B(a)P-induced animals showed a significant decrease in albumin (2.8 mg/dL) and total protein (6.25 g/dL) in comparison with the control group. Moreover, TQ plus B(a)P treatment led to an increased level of total protein (6.6 g/dL) and albumin (3.4 mg/dL) together, compared to the group treated with B(a)P only (Figure 2; *p* < 0.05).

### 2.3. Effect of Thymoquinone on MDA and NO Levels

**Figure 3:**
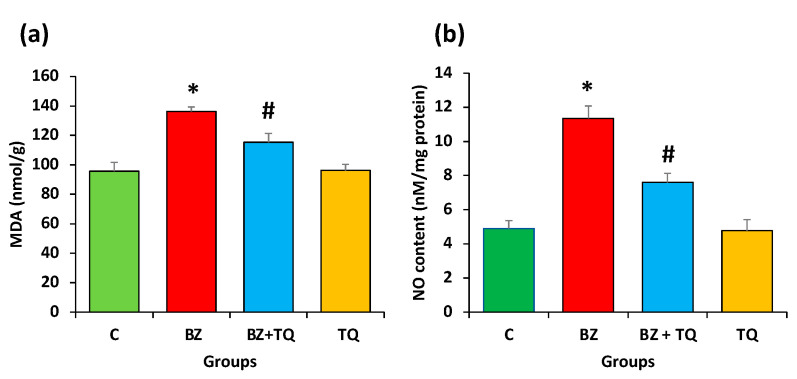
(**a**) The level of MDA was considerably increased in the animal group intoxicated with only B(a)P in comparison with the control group. TQ treatment significantly decreased the MDA level compared to the group treated with B(a)P only. (**b**) The NO concentration was considerably amplified in the group treated with B(a)P only compared to the control animals. TQ treatment decreased the NO level significantly in comparison with the B(a)P-treated group. The values denote the mean ± SEM, with 8 rats/group. The statistical differences are denoted with an asterisk (*), indicating significance at *p* < 0.05 in comparison with the control group, and a hashtag (#), signifying *p* < 0.05 in comparison with the disease control.

The concentration of MDA was significantly increased in the B(a)P-treated group (136.3 nmol/g) compared to the control groups (95.7 nmol/g) (*p*  <  0.05). In TQ-treated groups, MDA concentration was significantly reduced (115.4 nmol/g) compared to the B(a)P group (*p*  <  0.05). (Figure 3a). These findings demonstrated that B(a)P promotes potential oxidative damage in lungs.

Moreover, B(a)P notably increased nitric oxide (NO) levels compared to the control group (Figure 3b). However, TQ at 50 mg/kg b.w. considerably decreased the NO levels (*p* ≤ 0.05).

### 2.4. Effect of Thymoquinone on Antioxidant Enzyme Levels

**Figure 4:**
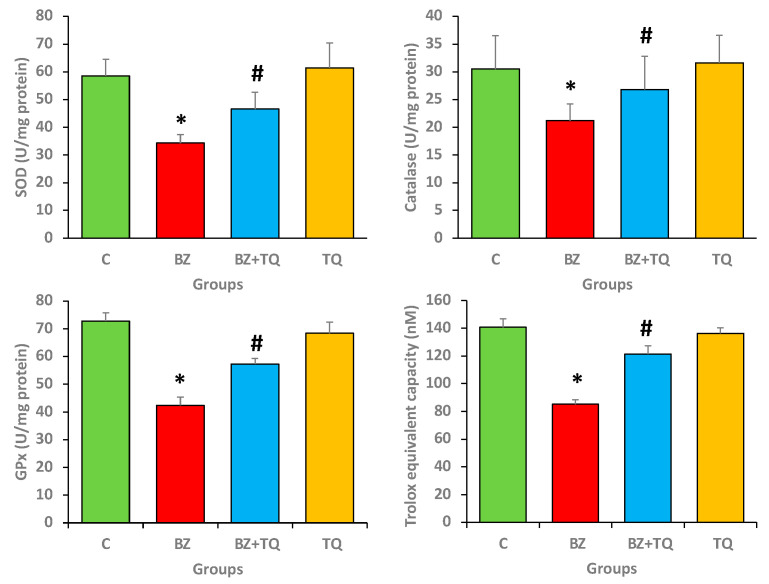
Catalase, SOD, GPx, and overall antioxidant capacity were considerably reduced in the group treated with only B(a)P in comparison with the control group. The activities of these enzymes significantly recovered after treatment with TQ. The numbers signify the mean ± SEM, with 8 rats/group. The statistical differences are denoted with an asterisk (*), indicating significance at *p* < 0.05 in comparison with the control group, and a hashtag (#), signifying *p* < 0.05 in comparison with the disease control.

The administration of benzopyrene significantly reduced the levels of various antioxidant enzyme markers. Compared to the control rats, B(a)P-treated rats exhibited a significant decrease in the levels of SOD, CAT, and *GPx* compared to the control animals. The TQ at 50 mg/kg b.w. promoted a substantial increase (*p* < 0.05) in all these antioxidant enzyme levels (Figure 4) compared to the B(a)P only administered group.

The effect of TQ on total antioxidant capacity was further evaluated. B(a)P treatment clearly reduces the total antioxidant capacity in comparison with the normal control animals. The TQ treatment (50 mg/kg b.w.) exhibited a significant increase (*p* < 0.05) in total antioxidant capacity (Figure 4) compared to the group intoxicated with B(a)P only. These results clearly show that TQ performs a vital role in the enhancement of antioxidant enzymes as well as antioxidant capacity.

### 2.5. Effect of B(a)P and TQ on Anti-Inflammatory Markers

**Figure 5:**
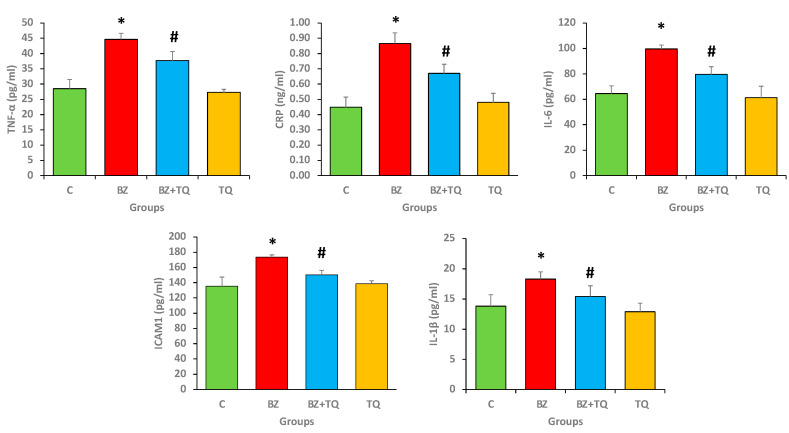
Inflammatory marker levels were significantly increased in animals intoxicated with B(a)P compared to the control group. B(a)P plus TQ treatment significantly decreased these inflammatory marker levels. The graphs represent the mean ± SEM, with 8 rats/group. The numbers signify the mean ± SEM, with 8 rats/group. The statistical differences are denoted with as asterisk (*), indicating significance at *p* < 0.05 in comparison with the control group, and a hashtag (#), signifying *p* < 0.05 in comparison with the disease control.

Benzopyrene significantly enhances the level of inflammatory cytokines such as TNF-α, IL-1β, IL-6, and ICAM1 in comparison with the control group. TQ (50 mg/kg b.w.) treatment significantly decreased all these inflammatory markers compared with the B(a)P-treated group (*p* < 0.05) (Figure 5).

### 2.6. Effect of TQ on Lung Architecture

**Figure 6:**
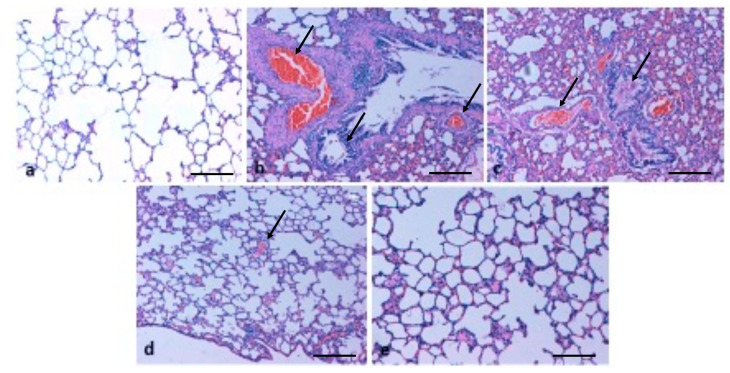
Histopathological changes of lung tissues in different animal groups. (**a**) Typical lung architecture in control rats includes normal airway bronchi and bronchioles and blood vessels and alveolar sacs. (**b**,**c**) B(a)P mediated the lung epithelium thickening, damage of the alveolar architecture, and infiltration of inflammatory cells within lung tissues, incidence of congestion and haemorrhage. The arrows indicate infiltration of inflammatory cells and congestion. (**d**) Histopathological changes were found to be considerably lower with TQ plus B(a)P co-administration, as this attenuated the damage to lung epithelium and alveolar architecture and decreased the incidence of edema. The arrow indicates less edema. (**e**) Thymoquinone-only treated group shows normal lung architecture (Scale bar = 100 μm).

H&E staining was used to study the lung tissue architecture in different animal groups by studying the defensive effect of TQ against B(a)P-induced lung injury. The lung tissues of control group rats showed normal histology, including normal airway bronchi, bronchioles, blood vessels, and alveolar sacs by microscopic observations. The administration of B(a)P in rats increased the thickening of lung epithelium, damaged the alveolar architecture, and enhanced the infiltration of inflammatory cells in the lung tissues. The incidence of congestion, haemorrhage and fibrosis was increased (Figure 6), whereas such histopathological changes were found to be considerably lower in the group co-administered TQ plus B(a)P, as TQ reduces the damage of lung epithelium and alveolar architecture, and lowers the infiltration of inflammatory cells, edema, and congestion within the lung tissues.

**Figure 7:**
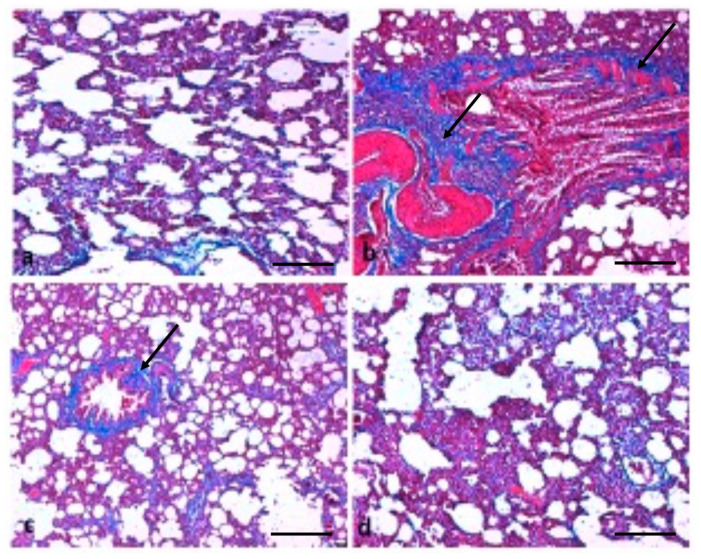
Effect of TQ on lung fibrosis. (**a**) Normal fibre architecture of lung in control animals; (**b**) B(a)P administration induces severe collagen deposition; the arrow indicates high deposition of collagen fibers (**c**) TQ shows suppression of B(a)P-induced fibrosis; the arrow indicates less deposition of collagen fibers. (**d**) normal collagen in the group treated with TQ only. (Scale bar = 100 μm).

The consequence of TQ on lung fibrosis was also measured by staining collagen fibres using Masson’s trichrome stain. The collagen staining showed that B(a)P administration induced severe collagen deposition (Figure 7); furthermore, such lesions were decreased by treatment with TQ. TQ administration showed suppression of B(a)P-induced fibrosis, as confirmed by Masson’s trichrome staining.

**Figure 8:**
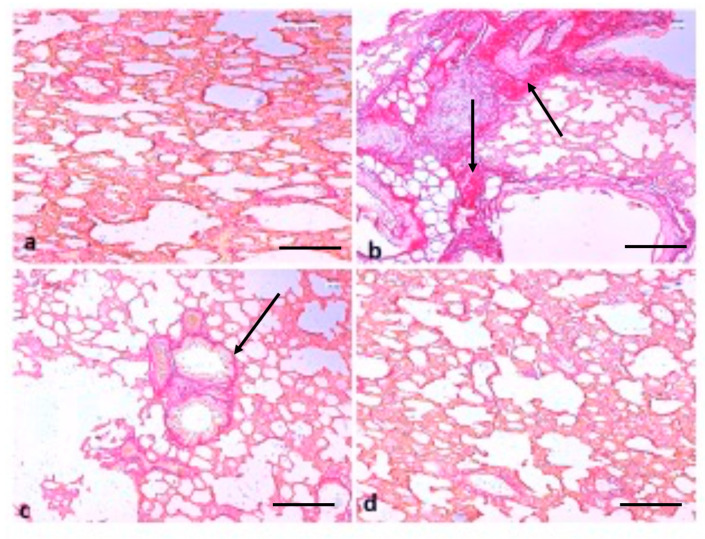
Effect of TQ on lung fibrosis. (**a**) Normal fibre architecture of lung in control animals; (**b**) B(a)P administration induces severe fiber deposition; the arrow indicates deposition of fibers (**c**) TQ shows suppression of B(a)P-induced fibrosis, the arrow indicates less deposition of fibers and (**d**) normal fibre architecture observed in the group treated with TQ only. (Scale bar = 100 μm).

In addition, Sirius red staining demonstrated that the fibrosis levels were more in B(a)P-treated lung tissue rats, whereas TQ administration group rats showed ameliorated fibrosis formation (Figure 8). These findings reveal that B(a)P-treated rat lung tissue causes accumulation of fibrosis and TQ exhibits a protective role against the lung damage and fibrosis formation caused by B(a)P.

## 3. TQ Reduces NF-κB Expression in Lung Tissue

**Figure 9:**
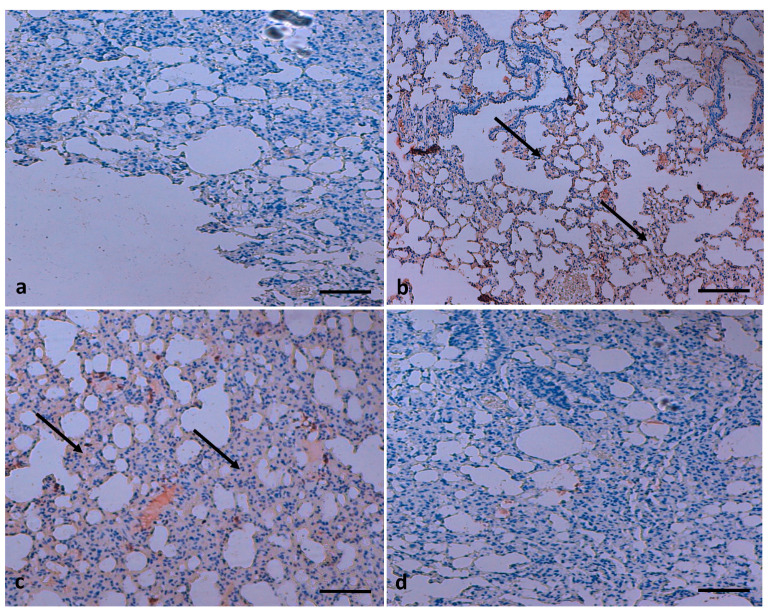
NF-κB protein expressional analysis. (**a**) The control rats did not express this protein; (**b**) the group treated with B(a)P only displayed high expression of NF-κB, the arrow indicates cytoplasmic positivity of NF-κB (**c**) the expression of this marker protein was reduced in animals co-treated with both B(a)P and TQ together, the arrow indicates cytoplasmic positivity of NF-κB (**d**) the animals treated with only TQ did not show any expression. (Scale bar = 100 μm.)

The excessive activation of macrophage plays a chief role in the development of lung injury. As is evident from ELISA results, TQ was found to have a key role in inhibiting NF-κB. Immunohistochemistry (IHC) staining demonstrated that a significantly high cytoplasmic NF-κB expression is found in the B(a)P-induced group compared to control rat tissue (Figure 9). NF-κB expression was considerably decreased in the B(a)P plus TQ treatment group in comparison with the group treated with B(a)P only. 

### Effect of TQ on Terminal Deoxynucleotidyl Transferase-Mediated dUTP Nick End Labelling (TUNEL)-Positive Cells

**Figure 10:**
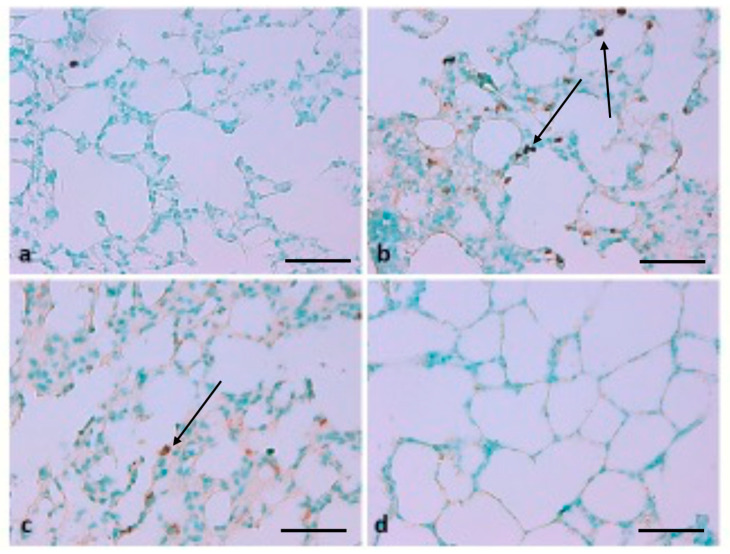
Effect of TQ on terminal deoxynucleotidyl transferase-mediated dUTP nick end labelling (TUNEL)-positive cells. (**a**) Brown-stained nuclei were not noticed in the control group; (**b**) large numbers of positive cells were stained brown in the B(a)P-induced lung injury group, the arrow indicates brown stained nuclei (**c**) the group treated with TQ plus B(a)P showed a significantly decreased apoptosis rate, the arrow indicates brown stained nuclei (**d**): the group treated with only TQ did not show any signs of apoptosis. (Scale bar = 100 μm.)

To evaluate the protective role of TQ in lung injury driven by B(a)P treatment, the TUNEL assay was performed in rat lung tissues. This staining showed that normal cell nuclei stain green, while these nuclei stain brown in apoptotic cells (Figure 10). Brown-stained nuclei were not noticed in the control group, while large numbers of positive cells were stained brown in the B(a)P-induced lung injury group. Positive cells in the TQ group were significantly diminished in comparison with B(a)P-induced group. The results further revealed that TUNEL-positive cells increased significantly in the B(a)P-induced group compared to the control group (Figure 10). Remarkably, compared with the B(a)P-induced group, the group treated with TQ showed a significantly decreased apoptosis rate. These results show that DNA fragmentation was significantly increased by B(a)P (*p* < 0.05), while TQ treatment significantly decreased DNA fragmentation.

## 4. Discussion

B(a)P, a well-known carcinogen, has been used in experimental animal models to cause lung injury and lung carcinogenesis. B(a)P causes alterations by dysregulating antioxidant enzymes, inflammation, and lung tissue architecture [21]. Rats intoxicated with B(a)P showed significantly higher triglyceride and total cholesterol levels. TQ at 50 mg/kg b.w. promoted a noticeable decrease in lipid profile compared to the group treated with B(a)P only. These results clearly indicate that TQ has a lipid-lowering effect.

The MDA level of the B(a)P (50 mg/kg b.w.) treatment group increased significantly relative to control groups. In TQ-treated groups, the MDA concentrations were reduced relative to the B(a)P group. These findings demonstrated B(a)P-mediated oxidative damage with increasing MDA concentration. Some previous studies are in agreement with the current study, and it was reported that a significant increase in the MDA level occurs in mice treated with only B(a)P compared with control animals [22]. The inflammatory response plays a significant role in fighting the interfering pathogens through the activation of specific immune cells such as macrophages and leukocytes [23]. Likewise, exposure to inhalator particles can lead to an inflammatory response within the lungs [24], and repeated exposure to such particles leads to tissue injury and chronic inflammation [25]. 

Respiratory tract inflammatory diseases are commonly linked with elevated production of nitric oxide (NO•) as well as increased indices of NO•-dependent oxidative stress [26]. In this study, it was observed that B(a)P significantly increased nitric oxide (NO) levels compared to the control group. With the administration of TQ at 50 mg/kg b.w., it significantly decreased the NO levels. In accordance with this finding, a previous study reported that NO generation was measured and it was found to be significantly increased after B(a)P treatment compared with the control group. Pretreatment with farnesol significantly reduced NO content in the BALF of the treatment group [27].

Overexpression of inflammatory cytokines provokes the development of lung pathogenesis, and the production of pro-inflammatory cytokines enhances the changes in lung cells. Here, in this study, it was noticed that B(a)P considerably increases the production of inflammatory cytokines. Treatment with TQ at 50 mg/kg b.w. significantly decreases all inflammatory markers compared to the animals treated with B(a)P only. The previous study explained the anti-inflammatory effect of thymoquinone and reported that IL-1β and IL-8 levels were elevated in the group exposed to cigarette smoke compared to the control group. Moreover, IL-8 levels declined in the group that received only TQ, which designated the anti-inflammatory effect of TQ [28]. Moreover, another study reported that TQ decreased the secretion of serum TNF-α and reduced the lung histopathological alternations induced by cyclophosphamide [29] and other cytokine levels [30].

TQ has been found to decrease the enhanced serum levels of OVA-specific IgE and IgG1. The histological analysis of lung tissue showed that TQ significantly decreases allergen-induced lung eosinophilic inflammation. In addition, TQ demonstrated a significant role in decreasing the production of IL-4, IL-5 and IL-13 and IFN-γ. Furthermore, TQ attenuates allergic airway inflammation through the inhibition of Th2 cytokines and eosinophil infiltration into the airways [31].

Antioxidants delay autoxidation by scavenging the species which initiate peroxidation reactions to produce reactive oxygen species (ROS) [32]. The controlled ROS formation and the endogenous antioxidant defence equilibrium are important in the inhibition of pathogenesis. Natural compounds as a whole or their specific active compounds are a rich source of antioxidants, and such natural antioxidants inhibit pathogenesis through scavenging ROS. Compared to the control animals, B(a)P-intoxicated rats exhibit a noticeable decrease in the antioxidant enzyme levels. TQ (50 mg/kg b.w.) promoted a substantial increase in the antioxidant status compared to the disease control group (the group administered B(a)P only). This finding clearly evidences that TQ plays a vital role in the enhancement of antioxidant enzymes. In this regard, previous studies have also reported that B(a)P treatment considerably lowers the activities of SOD and CAT compared to the control animals. Plant extract treatment appreciably restored the activities of these antioxidant enzymes in a dose-dependent manner [22]. Another study showed that B(a)P treatment decreases the level of SOD expressively in the B(a)P-administered group compared to control animals [33]. A pioneering study reported that TQ lowers lipid peroxidation and restores the antioxidant enzyme levels towards the normal values. Moreover, TQ possesses preventive and therapeutic potential against lung fibrosis through the inhibition of oxidative stress [34]. Another study reported that TQ treatment restores antioxidant enzyme activity towards normal values [35]. 

Here, in this study, it was noticed that B(a)P administration in rats led to the damage of alveolar architecture and promoted the infiltration of inflammatory cells, resulting in the incidence of congestion, whereas such histopathological changes were found to be considerably lower in the group co-administered TQ and B(a)P. Some previous studies have been reported to be in accordance with the current finding that treatment with only B(a)P results in necrosis and distorted lung architecture of the epithelium compared to the control animals. Moreover, treatment with plant extract protects against B(a)P-mediated lung injury [33]. 

The induction of lipopolysaccharide (LPS) causes pathological changes in the lung tissue of rats. The treatment with TQ has shown a significant role in the improvement of such pathological changes in these tissues. In addition, TQ shows a protective effect on LPS-caused inflammation in the lung and pathological changes in the lung tissue, which suggests the therapeutic role of TQ in lung injury [36]. 

Another study based on B(a)P treatment resulted in the thickening of the lung epithelium compared to the control animals. Moreover, a mass infiltration of inflammatory cells was found in B(a)P-administered animals. The treatment with diosmin led to protection against B(a)P-induced lung tissue pathological changes [37]. In the present study, it was observed that B(a)P treatment induced severe collagen deposition; furthermore, such lesions were decreased by treatment with TQ. Additionally, Sirius red staining demonstrated that fibrosis levels were more in B(a)P-treated lung tissue rats, whereas fibrosis formation was found to be ameliorated in the TQ administration group rats. Another study demonstrated that control group rats revealed normal architecture, while lung cancer-induced animals demonstrated hyperproliferative cells with enhanced deposition of collagen. Pretreatment with capsaicin noticeably minimized the collagen accumulation, thereby preserving the near-normal architecture [38]. Some previous findings have shown that TQ-treated animals show a reduction in the thickening of interalveolar septa, interstitial inflammatory edema, inflammatory exudates in the lumens of airways, and alveoli of bronchial-associated lymphoid tissue [39].

It has been reported earlier that TQ treatment inhibits the inflammatory pulmonary responses, meaningfully decreasing peribranchial inflammatory cell infiltration, alveolar edema, alveolar exudate, alveolar septal infiltration, interstitial fibrosis, as well as necrosis formation in toluene-treated rats. Moreover, TQ treatment leads to a significant decrease in the activity of in situ recognition of apoptosis, inducible nitric oxide synthase (iNOS), and an increase in the expression of surfactant protein D [40]. 

The TUNEL assay staining demonstrated that normal cell nuclei were stained green, while the apoptotic cell nuclei were stained brown. The TUNEL-positive cells were amplified significantly in B(a)P-treated animals in comparison with the control animals. Remarkably, the B(a)P-induced group showed a significantly enhanced apoptosis rate compared to the TQ treatment group. A study has reported that natural compounds decrease the apoptosis and that DIE animals showed the highest amount of apoptosis, which was completely or partially avoided by eugenol [41]. Similarly, another study reported apoptotic cell predominance after B(a)P administration of spermatogonial cells, whereas co-treatment with curcumin and resveratrol led to a decreased number of TUNEL-positive cells [42].

The nuclear factor-κB (NF-κB) transcription factors regulate the gene expression of several physiological responses, including proliferation, inflammatory responses, differentiation, cell adhesion, and apoptosis [43]. In the current study, it was shown that the expression of NF-kB is an important inflammatory marker [44]. Higher cytoplasmic expression of NF-κB was noticed in the B(a)P-induced group compared to control rat tissue. NF-κB expression was significantly decreased in the group tread with B(a)P plus TQ compared to the group treated with B(a)P only. A previous study is in accordance with the current finding that higher expression of this protein is noticed in B(a)P-treated animals compared with control animals. Similarly, enhanced expression of pro-inflammatory markers (NF-κB, IL-6 and COX-2) was noticed in B(a)P-treated mice compared to the vehicle-treated animals and diosmin decreased the pro-inflammatory marker expression [37]. 

The concentrations of thymoquinone and benzo(o)pyrene in serum were not measured in different experimental groups; that is the limitation of this study. This approach may have further supported the antioxidant and anti-inflammatory roles of TQ against lung injury by reducing ROS generation.

## 5. Materials and Methods

### 5.1. Chemicals

Thymoquinone and benzopyrene were obtained from Sigma-Aldrich Chemical Co. (St. Louis, MO, USA). GPx, CAT, SOD, and total antioxidant capacity kits were purchased from Abcam Company (Abcam, Cambridge, UK). ELISA kits of rat CRP, IL-1β, IL-6, TNF-α, and ICAM-1 were also obtained from Abcam Company (Abcam, Cambridge, UK). Masson’s trichrome staining kits and Picro Sirius red staining kits were bought from the same company. In addition, antibody NF-κB was also purchased from the same company. All other reagents and chemicals used in this study were of high analytical grade. The conventional animal chow was obtained from a grain mill in Qassim, Saudi Arabia. 

### 5.2. Animals

Adult male Swiss albino rats (180–210 g) were purchased from King Saud University, Kingdom of Saudi Arabia. The animals were kept for one week to reduce the transportation stress and as an acclimatization period. They were housed as 8 rats/cage and fed with normal rat chow in a normal environment with 12 h light/12 h dark cycle and 45–65% relative humidity. The experiment was designed for 8 consecutive weeks.

After the sacrifice, the collected blood samples were allowed to clot in plain centrifuge tubes. The clotted blood was centrifuged at 3000 rpm for 10 min to separate the serum.

### 5.3. Animal Ethics

The guidelines of animal care according to the College of Applied Medical Sciences, Qassim University, were followed. The animal ethics committee of Qassim University approved this research project, and maximum efforts were made to minimize the suffering of animals.

### 5.4. Animal Grouping and Treatment Plan

The animals were divided into four groups randomly, each group having eight rats, as follows (Table 1). 

### 5.5. Measurement of Lipid Profile

The lipid parameters such as triglycerides (TG) and total cholesterol (TC) were measured with specific kits photometrically and the result was interpreted accordingly.

### 5.6. Malondialdehyde (MDA) and NO Contents

Thiobarbituric acid reactive substance (TBARS) formation was used to check the malondialdehyde (MDA) level, a marker of lipid peroxidation. Malondialdehyde (MDA) concentration was measured in the lung tissues by the MDA assay kit according to a previously reported method [46].

Briefly, TBARS reacts with MDA and forms a product, the absorbance of which is measured at 534 nm. Furthermore, the tissue NO levels were evaluated as previous described [47].

### 5.7. Measurement of Antioxidant Enzymes 

The lungs were rapidly removed, weighed, and homogenized in phosphate buffer solution. The lung homogenates were centrifuged for 15 min at 4 °C at 5000× *g*. The supernatants were collected and aliquoted at 4 °C for biochemical analysis.

### 5.8. Total Antioxidant Capacity Measurement

Total antioxidant capacity assay is used to measure either the combination of both small molecule antioxidants and proteins, or small molecule antioxidants only in the presence of a protein mask. The reduced Cu^+^ ion chelates formed in this assay gives a broad absorbance peak around 570 nm which is proportional to the total antioxidant capacity of a sample.

### 5.9. Measurement of Inflammatory Markers

The inflammatory markers like TNF-α, IL-6, IL-1β and ICAM1 were measured by ELISA kits to evaluate the inflammatory parameters according to the manufacturer’s protocol.

### 5.10. Haematoxylin and Eosin (H&E) Staining

To fix the lung tissues, 10% neutral buffered formalin solution was used, and the tissues were dehydrated in graded alcohol. The tissues were cleared in xylene, and paraffin was used to embed these tissues. The embedded tissues were cut into 5 µm thick sections with a Leica microtome. Haematoxylin and eosin (H&E) staining was performed to evaluate the lung tissue architecture and the photographs were captured and the results were interpreted accordingly. The lesion scores were achieved as described by earlier methods [48,49]. The lesions scores were represented as follows: 0 = no alterations, +1 = mild, +2 = mild to moderate, and +3 = severe or diffuse alterations.

### 5.11. Masson’s Trichrome Staining

The degree of fibrosis and accumulation of collagen were determined by utilizing Masson’s trichrome staining. The formation of blue stains results from the collagen accumulation. Two independent pathologists were consulted to evaluate the sections in a blinded manner. The light microscope (Nikon Corporation, Tokyo, Japan) with ×100 magnification was used to obtain Masson’s trichrome staining images and the results were interpreted accordingly. The criteria for collagen fibre scoring were evaluated as follows: 0, normal; 1, mild (small fibrous area); 2, moderate (increased fibrosis); 3, severe (large fibrous areas).

### 5.12. Picro Sirius Red Staining

Picro Sirius red staining is used to check the fibrosis. In this method, the tissue sections were deparaffinized with xylene and were hydrated. The tissues sections were completely covered by Picro Sirius red solution and incubated for 1 h. Acetic acid solution was used to rinse all the sections followed by rinsing with absolute alcohol. The slides were cleared and mounted with DPX and the results were interpreted accordingly.

### 5.13. Terminal Deoxynucleotidyl Transferase-Mediated dUTP-Biotin Nick-End Labelling (TUNEL) Assay

The apoptotic nuclei in lung tissues are assayed by the TUNEL assay kit. TUNEL-positive nuclei were evaluated and photographs of all slides were taken under a microscope and the results were evaluated accordingly.

### 5.14. Expressional Evaluation of *NF*-*κB* Protein

Immunohistochemistry was used to evaluate the expression pattern of NF-κB protein as per the method previously explained [50,51]. In brief, antigen retrieval was performed to unmask the antigen sites. The primary monoclonal antibodies against NF-κB were added to lung sections and incubated overnight at 4 °C temperature. Biotinylated secondary antibodies (Abcam kits, Cambridge, UK) were added for 45 min at room temperature. Thereafter, horseradish peroxidase (HRP)-conjugated streptavidin was applied for a further 35 min at room temperature. Diaminobenzidine (DAB) was used as chromogen and haematoxylin stain was used as a counterstain. The images were captured by a digital camera, and the results were interpreted accordingly.

### 5.15. Statistical Analysis

All values obtained were expressed as mean  ±  SD. The significance of the differences among groups was determined by Tukey’s test for multiple comparisons using one-way analysis of variance (ANOVA). *p*  <  0.05 was considered a significant difference between the animal groups.

## 6. Conclusions

The outcomes of the present study revealed that thymoquinone has potential antioxidant and anti-inflammatory effects and can protect the lungs against injury by reducing ROS generation. TQ shows its antioxidant potential by enhancing antioxidant enzymes such SOD, catalase, and GPx and also improves the total antioxidant capacity. In addition, TQ reduces inflammatory markers such as TNF-α, IL-6, IL-1β, and ICAM1 and inhibits lung pathogenesis. TQ also restores the serum albumin and total protein content. Moreover, TQ plays a significant role in the maintenance of the architecture of lung tissue by attenuating the damage to lung epithelium and alveolar architecture. TQ also decreases the lung fibrosis formation and decreases DNA fragmentation. Overall, the current findings suggest that TQ can be used as a lung-protective agent against benzopyrene-induced lung injury. The limitation of this study was not measuring the concentrations of TQ and B(a)P in the serum of different experimental groups to support the antioxidant and anti-inflammatory properties of TQ further.

## Figures and Tables

**Table 1:** Animal grouping and treatment plan.

Group Number	Short Name	Description
Group I	C	The rats received normal saline solution
Group II	BZ	B(a)P in corn oil (50 mg/kg b.w.) was administered orally [45] thrice a week for 8 consecutive weeks
Group III	BZ + TQ	Co-treatment with B(a)P (50 mg/kg b.w.) and TQ (50 mg/kg b.w.), where TQ was given orally before B(a)P administration
Group IV	TQ	The rats received TQ (50 mg/kg b.w.) three times/week

## Data Availability

The data used to support the findings of this study are included within the article.
